# Genome-Based Drug Target Identification in Human Pathogen *Streptococcus gallolyticus*

**DOI:** 10.3389/fgene.2021.564056

**Published:** 2021-03-25

**Authors:** Nosheen Afzal Qureshi, Syeda Marriam Bakhtiar, Muhammad Faheem, Mohibullah Shah, Ahmed Bari, Hafiz M. Mahmood, Muhammad Sohaib, Ramzi A. Mothana, Riaz Ullah, Syed Babar Jamal

**Affiliations:** ^1^Department of Bioinformatics and Biosciences, Capital University of Science and Technology, Islamabad, Pakistan; ^2^Department of Biological Sciences, National University of Medical Sciences, Rawalpindi, Pakistan; ^3^Department of Biochemistry, Bahauddin Zakariya University, Multan, Pakistan; ^4^Department of Pharmaceutical Chemistry, College of Pharmacy, King Saud University, Riyadh, Saudi Arabia; ^5^Department of Pharmacology, College of Pharmacy, King Saud University, Riyadh, Saudi Arabia; ^6^Department of Soil Science, College of Food and Agriculture Sciences, King Saud University, Riyadh, Saudi Arabia; ^7^Department of Pharmacognosy (MAPPRC), College of Pharmacy, King Saud University, Riyadh, Saudi Arabia

**Keywords:** *Streptococcus gallollyticus*, infective endocarditis, pan-genome, subtractive proteomics, drug prioritization

## Abstract

*Streptococcus gallolysticus* (*Sg*) is an opportunistic Gram-positive, non-motile bacterium, which causes infective endocarditis, an inflammation of the inner lining of the heart. As *Sg* has acquired resistance with the available antibiotics, therefore, there is a dire need to find new therapeutic targets and potent drugs to prevent and treat this disease. In the current study, an *in silico* approach is utilized to link genomic data of *Sg* species with its proteome to identify putative therapeutic targets. A total of 1,138 core proteins have been identified using pan genomic approach. Further, using subtractive proteomic analysis, a set of 18 proteins, essential for bacteria and non-homologous to host (human), is identified. Out of these 18 proteins, 12 cytoplasmic proteins were selected as potential drug targets. These selected proteins were subjected to molecular docking against drug-like compounds retrieved from ZINC database. Furthermore, the top docked compounds with lower binding energy were identified. In this work, we have identified novel drug and vaccine targets against *Sg*, of which some have already been reported and validated in other species. Owing to the experimental validation, we believe our methodology and result are significant contribution for drug/vaccine target identification against *Sg*-caused infective endocarditis.

## Introduction

*Streptococcus gallolyticus* (*Sg*) is Gram-positive, non-motile bacteria previously referred as *Streptococcus bovis*. It is phenotypically diverse bacteria belonging to the Lancefield Group D Streptococci (Pasquereau-Kotula et al., 2018; Arjun et al., 2020). This bacterium grows in chain or pairs and is non-γ-hemolytic or slightly γ-hemolytic but sometimes shows alpha-hemolytic activity on ovine blood agar plates (Rusniok et al., 2010; Hensler, 2011). Although commonly present in microflora, approximately 2.5–15% is present in the gastrointestinal tract of a healthy individual (Hinse et al., 2011) and become an opportunistic pathogen causing various diseases, including infective endocarditis, colon cancer, meningitis, and septicemia.

This opportunistic pathogenesis of *Sg* is dependent on genes involved in polysaccharide production, glucan mucopolysaccharide, a putative component of biofilm produced by this species, and three types of pili and collagen-binding protein (Takamura et al., 2014). These genes provide protection from host immune system and help in adherence to the epithelial lining of the heart (Rusniok et al., 2010), causing infection and resulting in endocarditis (Millar and Moore, 2004).

For the last two decades, a significant rise in incidence of infective endocarditis were observed worldwide (Tripodi et al., 2005; Marmolin et al., 2016; Shahid et al., 2018; Arregle et al., 2019; Chamat-Hedemand et al., 2020). Among 100,000 population, 2.6–7 cases of endocarditis have been reported per year, a significant proportion of which was contributed by streptococcal infections: with incidence of 17% in North America, 31% in other European countries, 39% in the South America, and 32% in rest of the world (Holland et al., 2016). This disease mostly occurs in elderly patients (Firstenberg, 2016), and the median age of patients is ≥58 (Vilcant and Hai, 2018). The risk of developing *Sg* endocarditis rises with the consumption of uncooked meat or fresh dairy products, weakened immune system, history of hepatic diseases, and comorbidities such as diabetes mellitus and rheumatic disorders (Cãruntu et al., 2014).

In the presence of primary infection, metabolic disorder, or immune-compromised state, *Sg* tries to cause endocardial injury. This injury then triggers the thrombus formation by the removal of fibrin and platelets. After thrombus formation, the bacteria enters into the bloodstream through the thrombus. As *Sg* has virulence properties, it can enter into the bloodstream in a paracellular manner without inducing major immune response and adheres to the damaged collagen-rich surface of the cardiac valve (endocardium). Once it is attached to the endocardium, this bacterium proliferates and forms a biofilm, which causes the inflammation in the lining of the heart and causes endocarditis (McDonald, 2009; Hensler, 2011).

Antibacterial drugs such as Penicillin G along with Gentamycin and estreptomicin are preferred medical treatments against infective endocarditis. Other options include Gentamicin-related Ceftriaxone and vancomycin in patients allergic to penicillin (Satué-Bartolomé and Alonso-Sanz, 2009). For patients with persistent fever and resistance to medical therapy, an expensive surgical intervention may be needed (Grubitzsch et al., 2016). *Sg* is resistant to penicillin, and one of the strains of *Sg* is also found to be resistant to tetracycline (Hinse et al., 2011). Therefore, development of an efficient treatment strategy against endocarditis, novel therapeutic targets, and potent drugs are urgently required.

For the rapid identification, many computational methods have been established such as core genome and subtractive genomic approaches that allow us to identify the core essential genomes and which do not possess any homology with the human genome (Caputo et al., 2019). These approaches has been used in a number of human pathogens such as *Corynebacterium diphtheria* (Jamal et al., 2017), *Corynebacterium pseudotuberculosis* (Tiwari et al., 2014), and *Treponema pallidium* (Jaiswal et al., 2017). This study is designed with a goal to exploit *in silico* approaches to link *Sg* species genomic data with its proteome and to identify the putative therapeutic targets. It can be used to classify potent inhibitors that may contribute to the discovery of compounds that can inhibit pathogenic developments (Jamal et al., 2017). The proteomes from the seven genomes of *Sg* were compared using a pan genome approach, from which only those genes were selected that were present in all the strains of *Sg* (Hinse et al., 2011). Then, the predicted core genome was further filtered out on the basis of essentiality for the bacteria, from which only 18 proteins were found to be essential, and all these proteins were non-homologous to the host (human). Out of these 18 proteins, 12 cytoplasmic proteins were identified as drug targets. These essential and non-host homologous protein targets were subjected to virtual screening using a library of 11,993 compounds. The identified putative targets might be used to design peptide vaccines and suggest novel lead druggable compounds that could bind to the proposed target proteins (Barh et al., 2011; Jamal et al., 2017; Uddin et al., 2019).

## Materials and Methods

### Genome Selection

In the current study, all available strains of *Sg* with available complete genome were considered for the pan genome analysis. A total of seven strains of *Sg* were selected; gene and protein sequences were retrieved from NCBI^1^.

### Identification of Core Genomes

The core genome of *Sg* was identified from pan genome analysis using EDGAR software (Blom et al., 2016). Only those genes that were common in all the strains of *Sg* were selected. The selection criteria in EDGAR software were as follows: one strain is selected as a reference strain, and rest of all the strains were compared with the reference strains and from which the core genomes were selected that were common in all the strains. The algorithm that it used was protein Basic Local Alignment Search Tool (BLASTp) with the standard scoring matrix BLOSUM62 and cutoff value of *E* = 1 × 10^–5^ (Blom et al., 2016).

### Identification of Non-host Homologous Proteins

The identified core genome of *Sg* was then subjected to BLASTp against the human proteome to find out the proteins non-homologous to human host using default parameters *e*-value = 0.0001, bit score ≥ 100, scoring matrix BLOSUM62 and identity ≥ 25%. Only those proteins that showed no hit against human proteome database were selected (Jamal et al., 2017).

### Identification of Essential Genes

The non-host homologous proteins were subjected to BLASTp against Database of Essential Genes (DEG) with the standard scoring matrix BLOSUM62, *e*-value = 0.001 and identity ≥25% to find out essential proteins that are indispensable for the survival of pathogen. The database of essential genes consist of experimentally validated data from eukaryotes, archaea, and prokaryotes, and it covers a large number of essential genes for 31 bacteria containing more than 12,000 bacterial essential genes (Luo et al., 2014).

### Drug Target Prioritization

For the determination of potential therapeutics, several factors are used like molecular weight, molecular function, cellular localization, pathway analysis, and virulence (Agüero et al., 2008). Molecular weight (MW) was determined by ProtParam tool^2^. Targets whose MW is <100 kDa are considered as best therapeutic target (Mondal et al., 2015). Molecular functions and biological process for target proteins were determined by Uniprot^3^. Subcellular localization of pathogen was performed by CELLO^4^. The cellular localization of bacteria determines the environment in which proteins operate. It affects the function of protein by controlling accessibility and availability of all types of molecular interaction partners. The knowledge of protein localization often plays an important role in characterizing the cellular function of hypothetical and newly discovered proteins (Scott et al., 2005). For pathway analysis, the Kyoto Encyclopedia of Genes and Genomes (KEGG) web tool^5^ was used to determine the role of protein targets in different cellular and metabolic pathways (Kanehisa and Sato, 2020). To identify virulence of protein targets, Virulence Factor Database (VFDB)^6^ was used, which determines the pathogenic virulence of the target proteins.

### Catalytic Pocket Detection

The shortlisted potential druggable proteins were further screened to detect the possible binding pockets by calculating the druggable score using DoGSiteScorer (Volkamer et al., 2012). It is an automated pocket detection tool that is used for the calculation of druggability of protein cavities. This tool needs sequence of interest in 3D structure format; therefore, SwissModel was used for the prediction of the 3D structure. SwissModel web tool predicts the 3D structures of protein targets (Nielsen et al., 2010). After obtaining 3D structures, the druggability evaluation was performed by DoGSiteScorer. This tool returns the pocket residue and druggability score, which ranges from 0 to 1. The score closer to 1 is considered as a highly druggable protein cavity (Jamal et al., 2017).

### Retrieval of Ligands

Eleven thousand nine hundred ninety-three druggable molecules with Tonimoto cutoff level of 60% were retrieved from the ZINC database (Sterling and Irwin, 2015). Then, partial charges were calculated, and energies of these compounds were minimized using energy minimization algorithm with default parameters. All minimized structures were saved in.mdb file. Then, these prepared ligands were used as an input file for molecular docking (Wadood et al., 2014).

### Validation of 3D Structures

All the 3D structures quality was further validated using RAMPAGE and ERRAT tool. RAMPAGE stands for RNA Annotation and Mapping of Promoters for the Analysis of Gene Expression. This tool does Ramachandran plot analysis and provides validity score for the 3D structure of target proteins. The score ≥80 were considered good (Batut and Gingeras, 2013). For further validation, ERRAT, an online tool, was used, which provides information about the protein structure with bad regions. The quality factor of the 3D structure ≥37% were considered good (Saddala and Adi, 2018).

### Preparation of Protein for Docking

The predicted 3D structures were further prepared for docking using the Molecular Operating Environment (MOE) tool. This tool is quite robust along with the meticulous algorithm. It not only predicts the top ranking poses but also prognosticate the root mean-square deviation (RMSD) along with the calculated energies of docked molecule (Pagadala et al., 2017). The 3D protonation and energy minimization of these 3D structures was done (Vilar et al., 2008); then, these minimized structures were further used as template for molecular docking.

### Molecular Docking of Drug Targets

**FIGURE 1:**
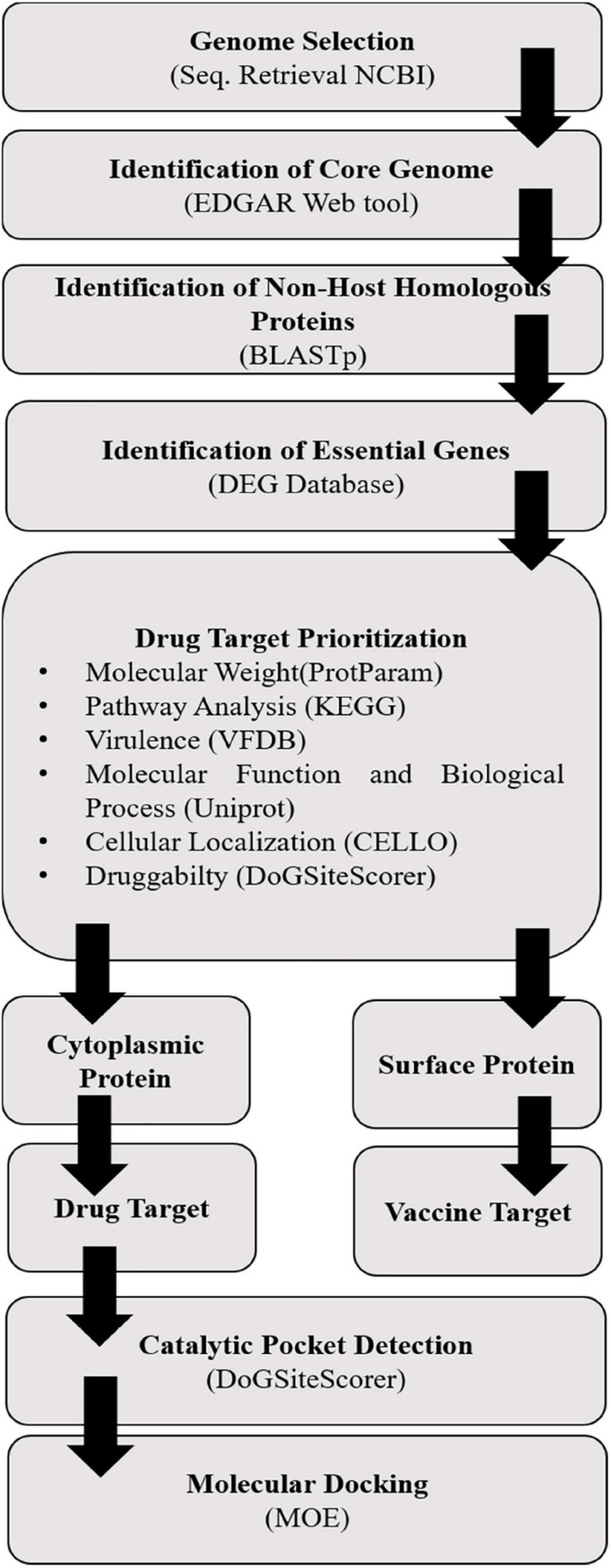
Complete workflow of drug target identification in *Sg* using *in silico* approaches.

The prepared minimized structures of targeted proteins and ligands were further subjected to molecular docking carried out in MOE using the MOE Dock (Figure 1). It predicted the favorable binding possess of selected ligands active sites of drug targets. Default parameters were selected for molecular docking. After the docking, we analyzed the best poses for hydrogen bonding/π–π interactions, and then, RMSD was calculated in MOE (Wadood et al., 2014). The orientation of the best dock molecules was further analyzed in chimera.

## Results and Discussion

### Genome Selection

The seven strains of *Sg* were retrieved from the National Center for Biotechnology Information (NCBI)^7^. The selection was based on the availability of their complete genome to have accuracy in our result. The details of the selected strains are summarized in Table 1.

**TABLE 1:** Strains of *Streptococcus gallolyticus* with information on genome statistics and regions of isolation.

Strains	Genome sizes (MB)	GC%	Total genes	Total proteins	Regions
DSM 16831	2.4929	37.70	2,498	2,341	Australia
NCTC13773	2.49358	37.70	2,496	2,333	Australia
ATCC 43143	2.36224	37.50	2,357	2,229	–
ATCC BAA-2069	2.37721	37.60	2,377	2,218	Germany
UCN34	2.35091	37.60	2,345	2,215	–
ICDDRB-NRC-S1	2.0525	37.70	2,125	1,759	Bangladesh
NCTC8133	1.86767	37.50	1,845	1,733	–

### Identification of Core Genomes

Core genome was identified to find drug targets that are homologous to all strains. Basically, only those core genomes that were defined as genes persistently present in all population of an organism were extracted (Uddin and Jamil, 2018). Core genomes were identified from pan genome analysis using EDGAR software. UCN34 strain was selected as reference genome, and the rest of the strains were compared to the reference strain. The total genes identified in pan genome were 3,242, out of which 1,138 were core genes.

### Identification of Non-host Homologous Proteins

The file generated by NCBI-BLASTp of *Sg* core genomes against human was parsed. Amidst 1,138 of core genomes, 1,115 proteins showed no hit and hence selected as non-homologous to the human proteome to avoid the aftereffect.

#### Identification of Essential Genes

The core 1,115 non-host homologous proteins were subjected to BLASTp against essential proteins present in DEG (Luo et al., 2014). The number of non-homologous proteins that is essential for the survival of the pathogen was 176. Among these, 18 proteins were selected as potential drug targets whose percent identity was >25, shown in Table 2. Out of these 18 proteins, 12 cytoplasmic proteins were selected as potential drug targets. The selection of final set of drug targets was kept strict to percentage identity to host, essentiality, and cutoff values.

**TABLE 2:** List of pathogen-essential non-host homologs proteins.

Query ID	Subject ID	% Identity	Proteins
GALLO_RS00005	DEG10330356	92.857	Chromosomal replication initiator protein DnaA
GALLO_RS00200	DEG10200056	80.769	Glucan-binding protein C
GALLO_RS00610	DEG10010101	54.688	Membrane protein insertase YidC
GALLO_RS00675	DEG10380051	53.659	Transcriptional regulator CtsR
SGGBAA2069_ RS00890	DEG10280041	51.448	PTS fructose transporter subunit IIA
GALLO_RS00830	DEG10470198	50	Penicillin-binding protein 2A
SGGBAA2069_ RS01250	DEG10180105	47.283	AraC family transcriptional regulator
GALLO_RS01215	DEG10110082	45.455	DNA polymerase III subunit alpha
GALLO_RS01760	DEG10060346	44	50S ribosomal protein L28
GALLO_RS01960	DEG10470004	41.793	2-isopropylmalate synthase
GALLO_RS02145	DEG10080178	40.355	Ribosome-binding factor A
GALLO_RS02350	DEG10050423	39.623	Amino acid ABC transporter substrate-binding protein, PAAT family/amino acid ABC transporter membrane protein, PAAT family
GALLO_RS02740	DEG10300014	38.71	DNA-binding response regulator
GALLO_RS02995	DEG10430209	38.197	16S rRNA methyltransferase B
GALLO_RS03395	DEG10180247	36.364	Glutamine ABC transporter permease
GALLO_RS03550	DEG10450136	35.789	Penicillin-binding protein 2B
GALLO_RS03570	DEG10460377	35.294	UDP-N-acetylmuramoyl-tripeptide–D-alanyl-D-alanine ligase
GALLO_RS03600	DEG10050249	35.135	1-acyl-sn-glycerol-3-phosphate acyltransferase

### Drug Target Prioritization

To determine the potential therapeutic targets, various factors were considered, including molecular weight from ProtParam (ExPASy, 2020; ProtParam documentation) of all 12 proteins was <100 kDa; therefore, these molecules featured as “druggable” molecule (Hughes et al., 2011). All these druggable molecules were analyzed using BLASTp against virulence factor database of VFDB (Chen et al., 2005), which predicts all therapeutics targets as virulent. Subcellular localization is a key factors in determining the function of protein. The CELLO (Yu et al., 2006) was used to predict the subcellular localization of 12 query proteins. These query proteins were further subjected for pathway analysis using KEGG database (Kanehisa et al., 2017). It appeared that most of the proteins are involved in metabolic pathways like enzymes, glycosyltransferases, peptidoglycan biosynthesis, degradation of proteins, and lipids biosynthesis proteins. Whereas a few of them are involved in cell signaling and cell processing such as secretion system and two-component system, very few proteins were involved in genetic information processing and resistance pathway such as transcription factor, ribosomes, DNA replication protein, mitochondrial biogenesis, β-lactam pathway, and vancomycin resistance pathway. The details about the drug target prioritization parameters and functional annotation of 12 essential non-host homologous proteins are shown in Table 3.

**TABLE 3:** Drug and vaccine target prioritization parameters and functional annotation of 12 essential non-host homologous proteins.

Uniprot ID	Protein	Gene	Biological function^*a*^	Molecular function^*b*^	Subcellular localization^*c*^	Virulent^*d*^	Molecular weight^*e*^ (kDa/Da)	Pathway analysis^*f*^
A0A139R4E3	Chromosomal replication initiator protein DnaA	dnaA	ATP binding, DNA replication origin binding	DNA replication initiation, regulation of DNA replication	Cytoplasmic	Yes	51,401.48	Two-component system
F5WXJ0	Transcriptional regulator CtsR	ctsR	DNA binding	Regulation of transcription, DNA-templated	Cytoplasmic	Yes	7598.78	Transcriptional regulator of stress and heat shock response
A0A3E2SCT8	PTS fructose transporter subunit IIA	DW662_ 04200	Phosphoenolpyruvate-dependent sugar phosphotransferase system	–	Cytoplasmic	Yes	14,982.13	No hit
A0A380K3P1	Penicillin-binding protein 2A	pbp2A	–	Penicillin binding, Transferase activity, transferring acyl groups	Cytoplasmic	Yes	84,763.57	Beta-lactam resistance
A0A380K803	AraC family transcriptional regulator	melR	Transcription, transcription regulation	DNA-binding transcription factor activity, sequence-specific DNA binding	Cytoplasmic	Yes	31,811.17	No hit
A0A380K8Y7	DNA polymerase III subunit alpha	dnaE	DNA replication	3′–5′ Exonuclease activity, DNA-directed DNA polymerase activity, nucleic acid binding	Cytoplasmic	Yes	165,491.77	DNA replication, mismatch repair, homologous recombination
A0A060RG19	50S ribosomal protein L28	rpmB	Translation	Structural constituent of ribosome	Cytoplasmic	Yes	6883.21	Ribosome
D3HCJ2	2-Isopropylmalate synthase	leuA	lLeucine biosynthetic process	2-Isopropylmalate synthase activity	Cytoplasmic	Yes	33,415.6	Biosynthesis of secondary metabolites, 2-oxocarboxylic acid metabolism, biosynthesis of amino acids, valine, leucine, and isoleucine biosynthesis, pyruvate metabolism, metabolic pathways
F5WZ36	Ribosome-binding factor A	rbfA	Maturation of SSU-rRNA	–	Cytoplasmic	Yes	13,409.48	No hit
A0A139R8A5	DNA-binding response regulator	DW662_ 02135	Phosphorelay signal transduction system, regulation of transcription, DNA-templated	DNA binding	Cytoplasmic	Yes	23,939.71	No hit
A0A1S5WAD9	16S rRNA methyltransferase B	BTR42_ 02745	Regulation of transcription, DNA-templated	RNA binding, rRNA methyltransferase activity	Cytoplasmic	Yes	19,761.96	No hit
F5WZQ7	UDP-N-acetylmuramoyl-tripeptide–D-alanyl-D-alanine ligase	murF	Cell cycle, cell division, cell wall organization, peptidoglycan biosynthetic process, regulation of cell shape	ATP binding, UDP-N-acetylmuramoyl-tripeptide-D-alanyl-D-alanine ligase activity	Cytoplasmic	Yes	50,278.43	Vancomycin resistance, peptidoglycan biosynthesis, metabolic pathways, lysine biosynthesis

*^a,b^Uniprot tool was used to identify the molecular and biological functions.*

*^c^CELLO tool was used to identify the cellular localization of the identified drug targets.*

*^d^VFDB tool was used to check whether these identified targets are involved in pathogen virulence or not.*

*^e^ProtParam tool was used to identify the molecular weight of the targeted proteins.*

*^f^KEGG tool was used for the identification of molecular pathway.*

Quality factor of 3D structures of druggable proteins were further validated through RAMPAGE and ERRAT. Quality factor predicted by both tool was ≥80 and ≥37%, respectively, as shown in Table 4. This score shows that our protein 3D structures are good and could be prepared for docking.

**TABLE 4:** Validation score of models from RAMPAGE and ERRAT.

S. No	Protein name	ERRAT	RAMPAGE
1	16S rRNA methyltransferase B	90.6699	92.30%
2	PTS fructose transporter subunit IIA	88.0435	90.80%
3	50S ribosomal protein L28	74.0741	87.50%
4	Chromosomal replication initiator protein DnaA	93.6747	92.60%
5	Penicillin-binding protein 2A	93.6823	91.30%
6	DNA polymerase III subunit alpha	89.1	88.90%
7	AraC family transcriptional regulator	100	97.00%
8	DNA-binding response regulator	93.0693	92.00%
9	Transcriptional regulator CtsR	100	100.00%
10	Ribosome-binding factor A	100	96.90%
11	UDP-N-acetylmuramoyl-tripeptide–D-alanyl-D-alanine ligase	94.7248	94.20%
12	2-isopropylmalate synthase	92.766	94.90%

#### Docking

Docking was performed against 12 drug targets with 11,993 ZINC druggable compounds via MOE tool. Top 100 molecules were redocked into the binding pocket of target proteins, and finally, a set of top 10 molecules was selected. The orientation of best docked molecule was analyzed in Chimera.

##### Validation of docking

**FIGURE 2:**
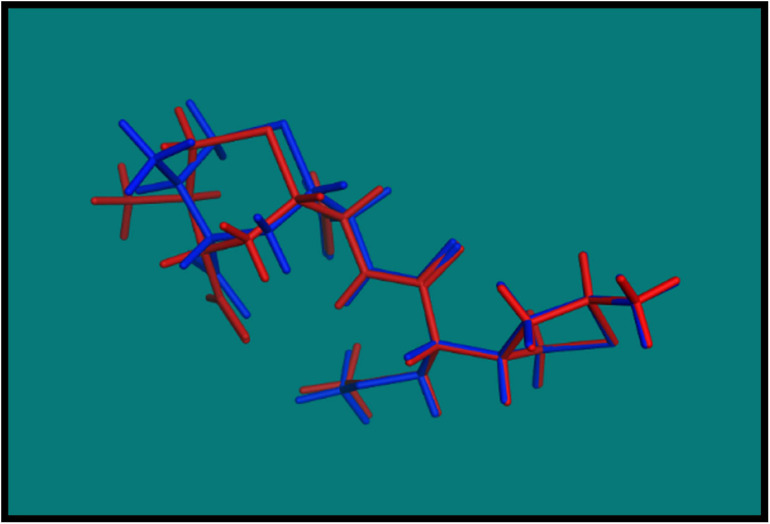
Blue native cocrystallized ligand and red dock ligand.

In order to validate the MOE Dock program, the cocrystallized ligand was removed from the active site and redocked within the inhibitor binding cavity of penicillin-binding protein (PDB ID: 3vsl). In this study, RMSD value (Figure 2) was found as 1.0968 Å, showing that our docking method is valid for the studied druggable molecules, and MOE Dock method, therefore, is reliable for docking of these compounds.

The analysis and biological significance of each of the predicted protein–ligand interaction are described as follows.

**FIGURE 3:**
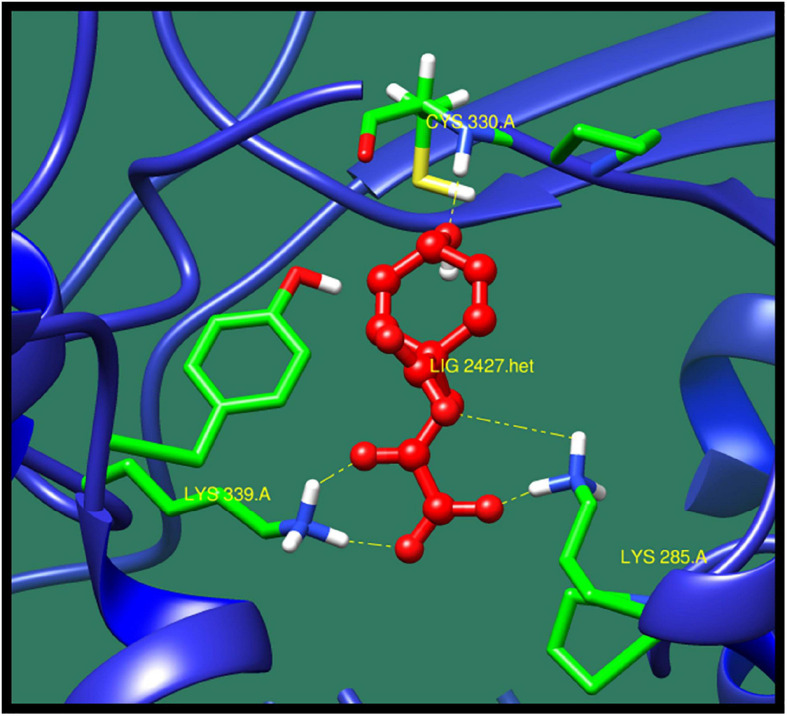
Interaction of 16S rRNA methyltransferase B with ZINC01532584 (colored in red). The interacting residues (green) are shown bonding (dotted lines) with the ligand.

*16S rRNA methyltransferase B* (*BTR42_02745*) is a protein that plays an important role in methylation of cytosine at position 967 of 16S rRNA. The structure of this protein consists of active sites in which two conserved cysteine residues are present. These cysteine residues are located near the activated methyl of cofactor. One of the cysteine residues act as a catalytic nucleophile and other play an important role in methyl transferase mechanism (Foster et al., 2003; Shen et al., 2020). The top 10 best confirmations are shown in Table 5 along with their ZINC ID, number of interactions, interacting residues, dock score, and minimized energy. The residues Lys 285, Lys 339, and Cys330 were found to interact with active ligand (ZINC01532584). The interaction of 16S rRNA methyltransferase B with ZINC01532584 is shown in Figure 3.

**TABLE 5:** 16S rRNA methyltransferase B and its interaction profile with docked compounds, their ZINC ID, minimized energy, number of interactions, dock score, and interacting residues.

ZINC ID	Number of interactions	Interacting residues	Minimized energy	Dock score
ZINC05835424	4	Ser 238, Asp327, Lys 263, Ala 328	–12.453	–13.4218
ZINC13650894	4	Lys 339, Lys 285, Lys 263, Asp 327	–14.373	–12.7997
ZINC13520246	3	Lys 263, Gly 262, Ser 238	–14.238	–11.2852
ZINC07001187	3	Arg 338, Asp 235, Lys 263	–18.2	–11.2818
ZINC32714665	4	Ala 328, Lys 263, Asp 327, Gly 262	–32.289	–12.2473
ZINC1404930	3	Tyr 282, Lys 285, Lys 339	–14.545	–11.9735
ZINC01711849	4	Lys 346, Lys 285	–0.952	–14.8757
ZINC01532584	5	Lys 285, Lys 339, Cys 330	–22.145	–11.7779
ZINC05181663	3	Ser 331, Lys 339, Lys 285	–21.977	–13.2929
ZINC44551376	5	Asp 341, Lys 339, Ser 27, Asn 28	–8.625	–12.3284

**FIGURE 4:**
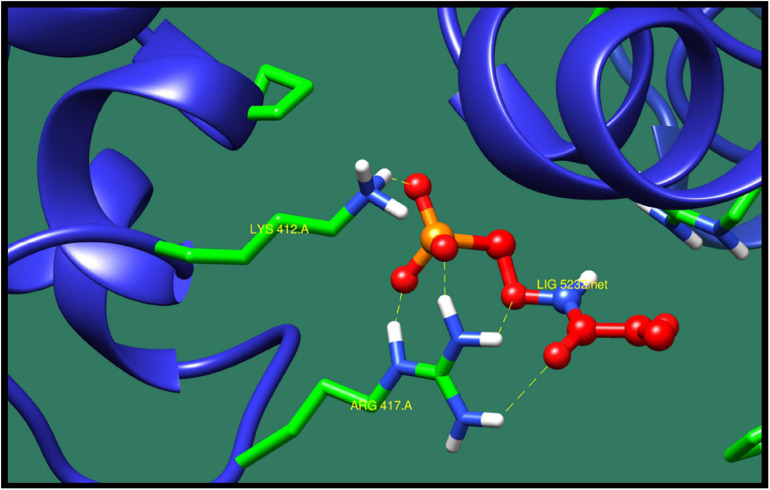
Interaction of chromosomal replication initiator protein DnaA with ZINC71782058 (colored in red). The interacting residues (green) are shown bonding (dotted lines) with the ligand.

*Chromosomal replication initiator protein DnaA* (*dnaA*) is a protein that plays a significant role in initiation and regulation of chromosomal replication. In DNA regulation, the initiation process is the key event in the cell cycle of all organism. The initiation of replication starts at the site of origin, which is recognized and processed by the initiator protein. The structure of this protein consist of nucleotide binding folds with the long helical connector to all-helical DNA binding domain. The conserved motif of this protein provide information about two most important steps in origin processing, which are binding of DNA and homo-oligomerization (Erzberger et al., 2002). Table 6 presents top 10 protein–ligand interaction with ZINC ID, minimized energy, number of interactions, Dock score, and interactive residues. ZINC71782058 was predicted as the most active lead compound against chromosomal replication initiator protein DnaA (*dnaA*). The protein–ligand interaction is shown in Figure 4.

**TABLE 6:** Chromosomal replication initiator protein DnaA and its interaction profile with docked compounds, their ZINC ID, minimized energy, number of interactions, dock score, and interactive residues.

ZINC ID	Number of interactions	Interacting residues	Minimized energy	Dock score
ZINC05839384	3	Lys 291, Asn 120, Lys 115	–12.715	–12.3924
ZINC07089629	2	Lys 291, Asn 120	–15.34	–16.1508
ZINC13540203	4	Arg 417, Lys 412, Asp 312	–21.772	–14.0893
ZINC71618824	2	Arg 417	–14.766	–11.6138
ZINC71782058	5	Arg 41, Lys 412	–24.383	–11.3505
ZINC72281564	3	Lys 291, Asn 120	–16.347	–17.7983
ZINC01585185	5	Lys 291, Asn 120, Lys 115	–12.005	–14.2479
ZINC01152242	2	Tyr 116, Lys 291	–13.191	–13.441
ZINC00387687	2	Lys 115, Glu 294	–0.384	–13.4207
ZINC01844424	2	Lys 291, Asn 113	–22.083	–12.841

**FIGURE 5:**
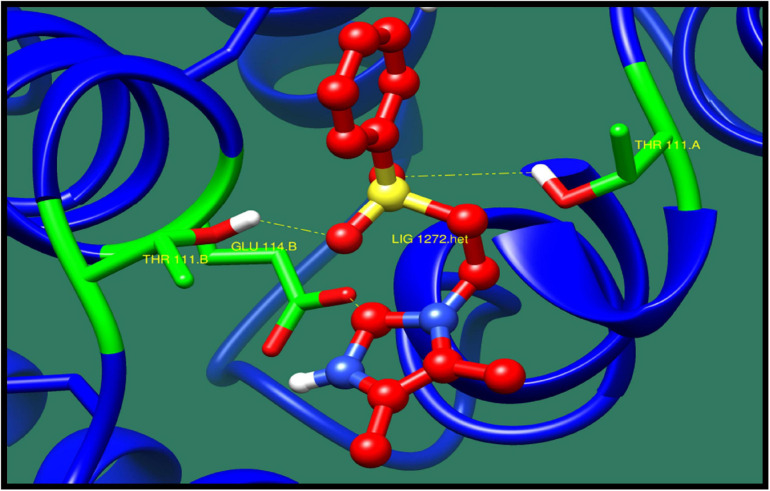
Interaction of transcriptional regulator CtsR with ZINC79090716 (colored in red). The interacting residues (green) are shown bonding (dotted lines) with the ligand.

*Transcriptional regulator CtsR* (*ctsR*) is an important repressor that regulates the transcription of class III stress genes in Gram-positive bacteria. CtsR controls the expression of genes encoding for chaperons and proteases. These genes play an important role in protein quality control system of bacteria. The structure of this protein consist of N-terminal DNA binding domain and C-terminal dimerization domain. N-Terminal DNA binding domain consists of helix-turn-helix (HTH) folds, and C-terminal dimerization domain consist of α-helices organized in four helix bundle. This protein also play an important role in pathogenicity, as it provides benefit to the bacteria during its stress condition and improves the survival chances for bacteria (Fuhrmann et al., 2009). Top 10 lead molecules against this protein are shown in Table 7 consisting ZINC ID, minimized energy, dock score, numbers of interactions, and interacting residues. The best interaction was shown by ZINC79090716 as shown in Figure 5.

**TABLE 7:** Transcriptional regulator CtsR and its interaction profile with docked compounds their ZINC ID, minimized energy, number of interactions, dock score, and interactive residues.

ZINC ID	Number of interactions	Interacting residues	Minimized energy	Dock score
ZINC05839384	3	Asp 124	–22.285	–12.3374
ZINC06962237	1	Thr 111	–20.134	–9.68175
ZINC19510011	2	Arg 113	–9.167	–9.14539
ZINC71603173	1	Glu 114	–79.985	–11.3391
ZINC77504434	1	Thr 111	–18.51	–9.38867
ZINC79090716	3	Thr A111, Thr B111, Glu 114	–37.17	–9.17204
ZINC01672834	1	Thr B111	–19.633	–9.02475
ZINC04352554	1	Thr B111	–9.073	–
ZINC655337127	2	Thr A111,Thr B111	–10.149	–9.23486
ZINC65337127	1	Thr A111	–20.417	–9.20724

**FIGURE 6:**
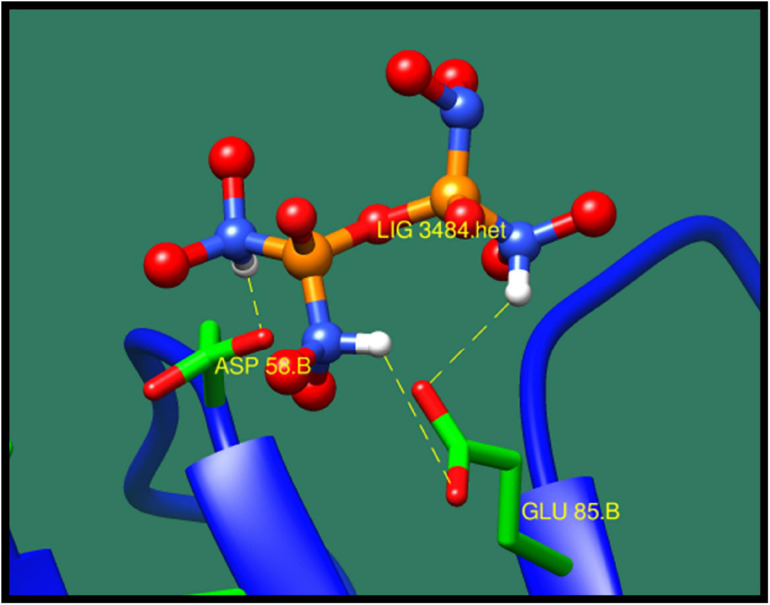
Interaction of phosphotransferase system (PTS) fructose transporter subunit IIA with ZINC01638334 (colored in red). The interacting residues (green) are shown bonding (dotted lines) with the ligand.

*Phosphotransferase system (PTS) fructose transporter subunit IIA* (*DW662_04200*) is a protein that is involved in phosphoenolpyruvate-dependent sugar PTS. In bacteria, it is a major carbohydrate transport system. PTS catalyzes the translocation with naturally occurring phenomenon of phosphorylation of sugar and hexitols, and it also regulates the metabolism in response to the availability of carbohydrates. It consists of two proteins HPr and enzyme I protein. These are the cytoplasmic proteins, in which first enzyme I transfers phosphoryl groups from phosphoenolpyruvate to phosphoryl carrying protein HPr. Then, this HPr further transfers the phosphoryl group to different transport complexes. PTS fructose transporter subunit IIA belongs to the fructose–mannitol family. This is a large and complex family that consists of several sequenced fructose and mannitol-specific permeases and putative permeases of unknown specificities. This family have three domains, IIA, IIB, and IIC, from which the most specific domain is IIA for the fructose PTS transporters (Siebold et al., 2001). The top 10 protein–ligand interaction is shown in Table 8, and the best interaction is shown in Figure 6 with ZINC01638334.

**TABLE 8:** Phosphotransferase system (PTS) fructose transporter subunit IIA and its interaction profile with docked compounds, their ZINC ID, minimized energy, number of interactions, dock score, and interactive residues.

ZINC ID	Number of interactions	Interacting residues	Minimized energy	Dock score
ZINC18033182	4	Asp 58, Glu 85	–52.033	–11.38848
ZINC32714665	3	His 83, Glu 85, Asp 58	–65.244	–11.38571
ZINC17004087	3	Glu 85, Tyr 87, Asp 58	–12.11	–11.27542
ZINC72145573	4	Lys 3, Glu 85, Asp 58	–19.982	–10.27034
ZINC71780811	4	Lys 118, Gln 28, Glu 22	–23.974	–9.37229
ZINC01638334	3	Asp 58, Glu 85	–27.139	–11.97846
ZINC01613419	4	Glu 85, Asp 58	–22.839	–11.70157
ZINC04261883	4	Glu 85, His 83	–11.661	–10.3571
ZINC38292458	3	Glu 85, Asp 58	–15.396	–10.78736
ZINC49625635	4	Asp 58, Glu 85	–51.252	–10.71723

**FIGURE 7:**
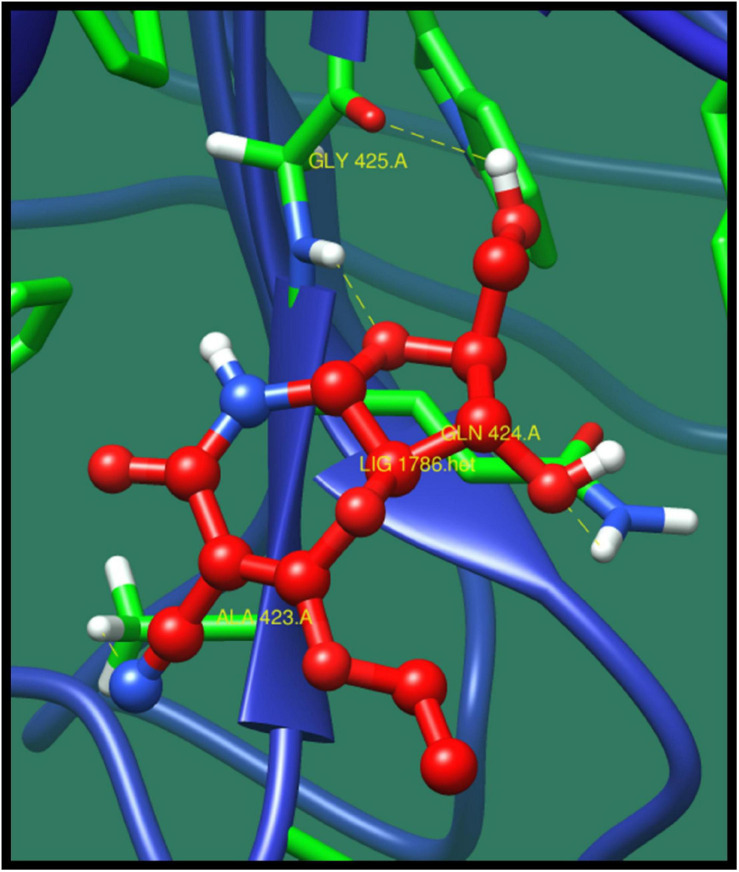
Interaction of penicillin-binding protein 2A with ZINC16942644 (colored in red). The interacting residues (green) are shown bonding (dotted lines) with the ligand.

*Penicillin-binding protein 2A* (*pbp2A*) is a transpeptidase that catalyzes the cell wall crosslinking, which is quite essential for the growth and survival of bacteria. This protein activation is regulated by active site at which the crosslinking take place (Fishovitz et al., 2014). Through pathway analysis, it is clear that it is involved in β-lactam resistance pathway. β-Lactam antibiotic is the most used group of antibiotics, which exerts its effect by interfering with the bacterial cell wall by structural crosslinking of peptidoglycan. This protein has already been reported as β-lactam resistant. This antibiotic resistance is due to the inactivation of the enzymes, change in β-lactam targets of pbp, change in porins, and use of efflux pump (Kocaoglu and Carlson, 2015). The top-ranked lead compounds are given in Table 9 where compound ZINC16942644 was predicted as best on the basis of minimized energy, dock score, and number of interactions made (Figure 7).

**TABLE 9:** Penicillin-binding protein 2A and its interaction profile with docked compounds, their ZINC ID, minimized energy, number of interactions, dock score, and interactive residues.

ZINC ID	Number of interactions	Interacting residues	Minimized energy	Dock score
ZINC05567030	2	Asp408	–13.81	–3.86291
ZINC22048956	3	Tyr 456, Glu 421, Gln 424	–15.356	–13.931
ZINC19799513	2	Lys 166, Asp 382	–19.047	–13.643
ZINC17004087	3	Asp 382, Glu 381	–16.385	–13.3505
ZINC18045201	3	Arg 443, Gln 424	–16.838	–13.1728
ZINC20502353	3	Tyr 456, Gln 424	–1.255	–13.1531
ZINC20070370	2	Gly 425, Ser 424	− −6.277	–12.7398
ZINC32628102	2	Arg 443, Gly 425	–13.827	–12.6254
ZINC16942644	4	Gln 424, Gly 425, Ala 423	–3.839	–12.581

**FIGURE 8:**
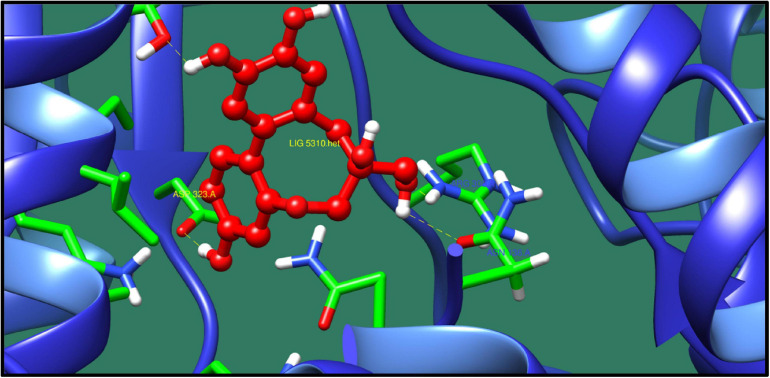
Interaction of UDP-N-acetylmuramoyl-tripeptide–D-alanyl-D-alanine ligase with ZINC14681317 (colored in red). The interacting residues (green) are shown bonding (dotted lines) with the ligand.

*UDP-N-acetylmuramoyl-tripeptide-D-alanyl-D-alanine ligase* (*murF*) is a protein involved in the biosynthesis of peptidoglycan. Peptidoglycan is the important component of bacterial cell wall, and enzymes involved in its synthesis could represent as potential drug target. MurF catalyzes the final step in the biosynthesis of the peptidoglycan in which it adds the D-Ala–D-Ala to the nucleotide precursor UDP-MurNAc-L-Ala-γ-D-Glu-meso-DAP (Hrast et al., 2013). The protein–ligand interaction of the top 10 molecules is shown in Table 10, and among these molecules, the best interaction was with ZINC14681317 as shown in Figure 8.

**TABLE 10:** UDP-N-acetylmuramoyl-tripeptide–D-alanyl-D-alanine ligase and its interaction profile with docked compounds, their ZINC ID, minimized energy, number of interactions, dock score, and interactive residues.

ZINC ID	Number of interactions	Interacting residues	Minimized energy	Dock score
ZINC14681317	5	Thr 338, Asp 162, Asp 323, Asn 36, Arg 308	–29.34	–13.2352
ZINC05842784	3	Asn 137, Asp 162, Glu 138,	–17.576	–12.9628
ZINC05811451	3	Thr 309, Asn 134, Arg 308	–12.09	–12.4832
ZINC32714665	3	Asn 162, Asn 137	–55.904	–13.7953
ZINC15768374	3	Asp 162	–14.941	–12.7596
ZINC71607274	3	Glu 138, Asp 162, Asn 137	–5.876	–13.2523
ZINC77323423	4	Arg 308, Asn 134, Thr 309, Thr 338	–7.009	–13.1429
ZINC73825281	4	Asp 162, Thr 338	–2.389	–13.0399
ZINC70503687	2	Thr 309, Thr 338	–41.806	–12.2618
ZINC55253127	3	Lys 130, Asp 116, Gly 133	–36.356	–10.7312

**FIGURE 9:**
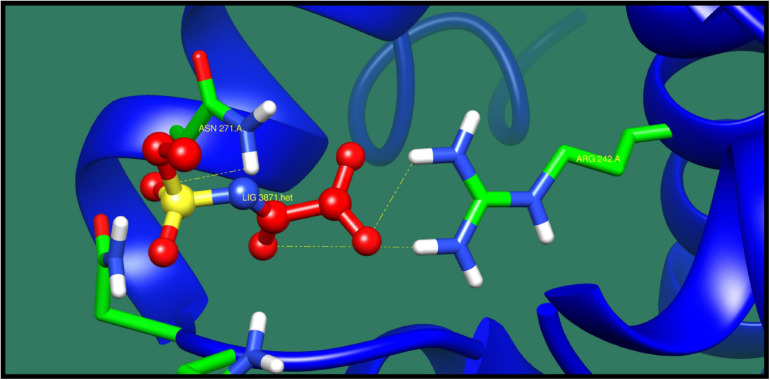
Interaction of AraC family transcriptional regulator with ZINC71781167 (colored in red). The interacting residues (green) are shown making bonding (dotted lines) with the ligand.

*AraC family transcriptional regulator* (*melR*) protein belongs to Arac/XylS family. This is a family of transcription regulators and is widely distributed in bacteria. This protein regulates the transcription of several genes and operons that are involved in arabinose catabolism and transport. This protein coregulates with another transcription regulator that is also involved in degradation of I-arabinose. By binding together, these regulators activate the transcription of five operons that are involved in transport, catabolism, and autoregulation of I-arabinose. Its structure is composed of C-terminal DNA binding domain and N-terminal domain. C-Terminal DNA binding domain consists of two HTHs that are connected with α-helix, and N-terminal domain is responsible for dimerization and binding of I-arabinose. The structure of this reveal that the N-terminal of this protein plays an important role in regulation of arabinose (Rodgers and Schleif, 2009; Fernandez-López et al., 2015; Malaga et al., 2016). Table 11 presents the best results against AraC family transcriptional regulator (*melR)* where ZINC71781167 was predicted as top lead compound as shown in Figure 9.

**TABLE 11:** AraC family transcriptional regulator and its interaction profile with docked compounds, their ZINC ID, minimized energy, number of interactions, dock score, and interactive residues.

ZINC ID	Number of interactions	Interacting residues	Minimized energy	Dock score
ZINC06691773	3	Arg 242	–1.55	–10.4279
ZINC13552228	3	Asp 248	–4.316	–8.72414
ZINC08627906	3	Asn 205, Ile 198	–10.822	–8.54297
ZINC15768388	2	Lys 245, Asn 205	–3.968	–11.6379
ZINC18164213	2	Tyr 202, Lys 245	–15.968	–10.3928
ZINC18141362	2	Asn 199, Val 241	–12.472	–10.4473
ZINC71603518	2	Asp 248	–6.232	–9.01625
ZINC71781167	3	Arg 242, Asn 271	–10.953	–8.5983
ZINC70632388	3	Arg 242, Gly 265, Asn 267	–10.747	–8.05192
ZINC71618824	2	Arg 242	–16.738	–8.03541

**FIGURE 10:**
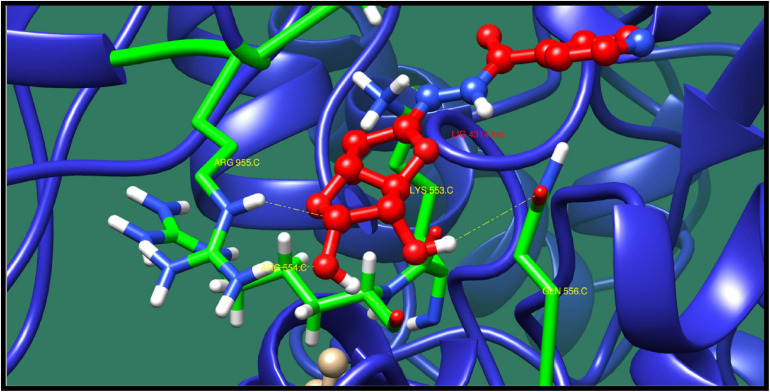
Interaction of DNA polymerase III subunit alpha with ZINC38653615 (colored in red). The interacting residues (green) are shown bonding (dotted lines) with the ligand.

*DNA polymerase III subunit alpha* (*dnaE*) is responsible for the replication in bacterial genome. This protein function as tripartite assembly consisting two core polymerases. In *Escherichia coli*, the core polymerases contain the catalytic α-subunit also known as PolIIIα, the 3′–5′ exonuclease ε-subunit and the θ subunit whose function is essentially unknown (Wing et al., 2008). From the function and pathway analysis, this protein is involved in DNA replication, mismatch repair pathway, and homologous recombination. It is located in the cytoplasm, which means it could act as drug target. The top 10 interaction of this protein with ligands is shown in Table 12 along with their ZINC ID, minimized energy, number of interactions, dock score, and interactive residues. The binding pocket residues Arg955, Lys553, Gln556, and Arg554 were predicted to contribute in the interaction with lead molecule ZINC38653615 as shown in Figure 10.

**TABLE 12:** DNA polymerase III subunit alpha and its interaction profile with docked compounds, their ZINC ID, minimized energy, number of interactions, dock score, and interactive residues.

ZINC ID	Number of interactions	Interacting residues	Minimized energy	Dock score
ZINC06566417	4	Arg 955, Arg 554, Gln 556	–17.247	–12.449
ZINC08616471	4	Asn 953, Arg 554, Gln 556, Lys 553	–15.181	–12.419
ZINC05766473	4	Arg 955, Arg 554, Gln 556	–22.601	–12.214
ZINC32599342	4	Asn 550, Leu 956, Lys 553, Gly 535	–15.541	–12.748
ZINC16248201	4	Gly 535, Leu 956, Lys 553	–17.411	–12.663
ZINC00351016	4	Asn 954, Gln 556, Asn 953, Arg 955	–18.342	–11.333
ZINC00440425	4	Lys 553, Arg 554, Asn 953	–23.546	–11.087
ZINC05204676	4	Lyss 538, Lys 919	–13.954	–12.305
ZINC38653615	7	Arg 955, Lys 553, Gln 556, Arg 554	–21.284	–14.76
ZINC44123372	3	Arg 955, Asn 953	–15.22	–11.411

**FIGURE 11:**
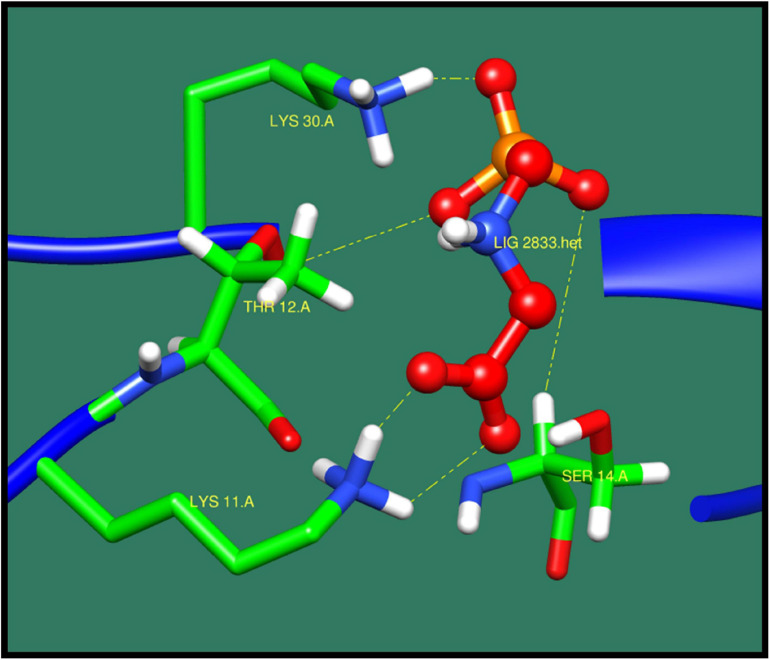
Interaction of 50S ribosomal protein L28 with ZINC03872713 (colored in red). The interacting residues (green) are shown bonding (dotted lines) with the ligand.

*50S ribosomal protein L28* (*rpmB*) protein plays an important role in the assembly of ribosome. This protein is encoded by rpmB operon. This protein could act as potential drug target as its role in ribosome assembly and functioning (Aseev et al., 2016). The functional analysis also showed its role in translation and structural constituent in ribosomes, which makes it a good drug target. The top 10 results of 50S ribosomal protein L28 protein is shown in Table 13 along with their ZINC ID, minimized energy, dock score, number of interactions, and interactive residues, and the best interaction was observed with ZINC03872713 shown in Figure 11.

**TABLE 13:** 50S ribosomal protein L28 and its interaction profile with docked compounds, their ZINC ID, minimized energy, number of interactions, dock score, and interactive residues.

ZINC ID	Number of interactions	Interacting residues	Minimized energy	Dock score
ZINC06691773	2	Lys 11	–0.723	–11.7263
ZINC13540203	4	Lys 11, Lys 30	–14.656	–10.0786
ZINC70632524	2	Ser 14, Lys 30	–7.156	–9.04594
ZINC77312688	3	Lys 11, Lys 30, Ser 14	–16.072	–8.63258
ZINC78442030	2	Trp 48, Ala 2	–7.335	–8.49688
ZINC01711849	4	Lys 11, Lys 30	–3.677	–13.9983
ZINC05372521	3	Lys 11, Ser 14	–4.936	–10.6346
ZINC03872713	5	Lys 11, Ser 14, Thr 12, Lys 30	–17.983	–9.81845
ZINC00053149	2	Lys 30, Ala 2	–5.882	–9.40368
ZINC03861035	2	Ala 2	–8.094	–8.84935

**FIGURE 12:**
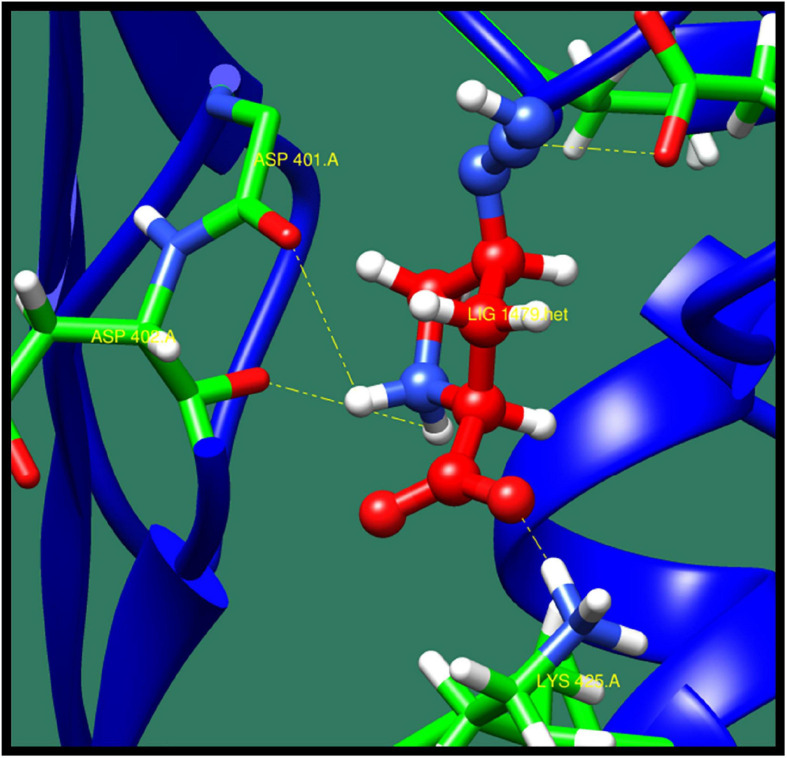
Interaction of 2-isopropylmalate synthase with ZINC40448986 (colored in red). The interacting residues (green) are shown bonding (dotted lines) with the ligand.

*2-Isopropylmalate synthase* (*leuA*) protein catalyzes to form 2-isopropylmalate by the condensation of acetyl group of acetyl-CoA with 2-oxoisovalerate. It is also involved in biosynthesis of leucine, by synthesizing L-leucine from 3-methly-2-oxobutanate (De Carvalho and Blanchard, 2006). In *Mycobacterium tuberculosis*, biosynthesis of leucine plays an essential role, which is important for the growth of bacteria, and so it could act as a potential drug target. The structure of this protein consist two domains N- and C-terminal. N-Terminal consist of triosephosphate isomerase (TIM) barrel catalytic domain, and C-terminal is a regulatory domain (Koon et al., 2004). The top 10 ligands against 2-isopropylmalate synthase (*leuA*) protein are shown in Table 14 along with ZINC ID, minimized energy, number of interactions, dock score, and interactive residue, and the best interacting protein–ligand confirmation is shown in Figure 12.

**TABLE 14:** 2-Isopropylmalate synthase and its interaction profile with docked compounds their ZINC ID, minimized energy, number of interactions, dock score, and interactive residues.

ZINC ID	Number of interactions	Interacting residues	Minimized energy	Dock score
ZINC 08939819	3	Asp 401, Lys 425, Asp 482	–11.066	–12.5294
ZINC05688692	3	Asp 401, Asp 482	–33.487	–10.2229
ZINC32714665	4	Lys 487, Asp482, Asp 401	–41.59	–14.2632
ZINC22056810	4	Asp 401, Asp 482, Ala 400, Asp 402	–7.075	–12.6048
ZINC83324781	3	Asp 482, Asp 401, Lys 425	–50.616	–10.6583
ZINC01235906	3	Asp 482, Lys 425	–10.86	–10.0718
ZINC40448986	5	Asp 401, Lys 425, Asp 402, Asp 482	–9.206	–15.485
ZINC49625635	4	Lys 425, Asp 401, Asp 482	–38.628	–14.7517
ZINC39134339	3	Asp 401, Asp 482, Lys 425	–12.214	–12.8497
ZINC38342322	4	Lys 425, Asp 401, Asp 402	–15.319	–12.2896

**FIGURE 13:**
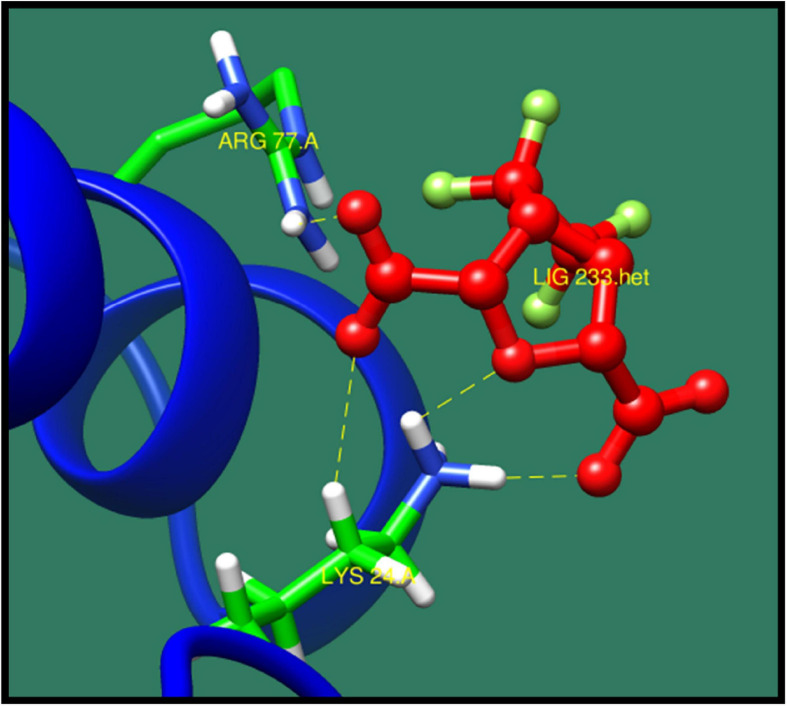
Interaction of ribosome-binding factor A with ZINC01235906 (colored in red). The interacting residues (green) are shown making bonding (dotted lines) with the ligand.

*Ribosome-binding factor A* (*rbfA*) is cold shock adaptation protein that helps bacteria to grow at low temperature (10–20°C). This protein associates with 30S ribosomal subunit but do not associate with 70S ribosomes or polysomes. It also interacts with 5′-terminal helix of 16S rRNA. During the cold shock adaptation, several cold shock proteins are synthesized, which allow the efficient translation processing of the messenger RNAs (mRNAs), which facilitates the ribosome assembly that is required for the growth of bacteria (Huang et al., 2003). This protein is found to be virulent and quite essential for bacteria so that it could act as potential drug target. The best interacting lead molecules are shown in Table 15 along with ZINC ID, minimized energy, dock score, number of interactions, and interacting residues. ZINC01235906 was predicted as top ranked molecule interacting with binding site residues lys24 and Arg77 (Figure 13).

**TABLE 15:** Ribosome-binding factor A and its interaction profile with docked compounds, their ZINC ID, minimized energy, number of interactions, dock score, and interactive residues.

ZINC ID	Number of interactions	Interacting residues	Minimized energy	Dock score
ZINC149388367	1	Lys 70	–14.308	–14.0562
ZINC83235996	2	Lys 24, Arg 77	–6.721	–11.0955
ZINC01235906	4	lys 24, Arg 77	–17.173	–11.1175
ZINC00171258	2	Lys 24, Arg 77	–10.793	–8.91597
zinc00255388	3	Arg 26, Lys 24	–6.936	–8.81042
ZINC01532584	4	Lys 24, Arg 77	–19.888	–8.78133
ZINC03872713	4	Arg 77, Arg 81, Lys 24	–15.475	–13.1602
ZINC05185127	3	Asp 27, Lys 63	–22.165	–10.6689
ZINC03852636	3	Lys 24, Arg 77, Arg 26	–10.595	–10.5473
ZINC58386852	3	Thr 74, Lys 24, Arg77	–10.798	–10.7004

**FIGURE 14:**
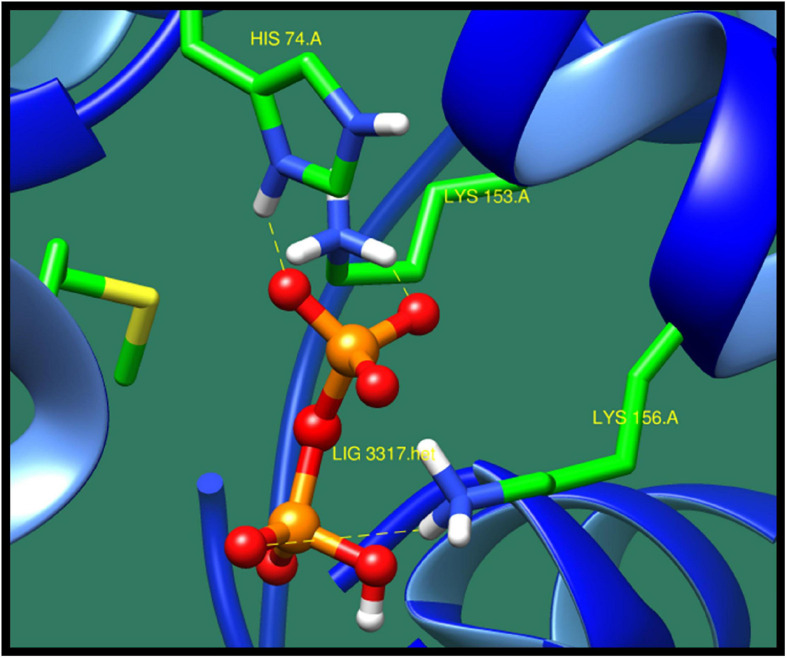
Interaction of DNA-binding response regulator with ZINC38140720 (colored in red). The interacting residues (green) are shown bonding (dotted lines) with the ligand.

*DNA-binding response regulator* (*DW662_02135*) is a protein that mediates the change in cell according to the response in the environment. This protein is a part of a two-component regulatory system (TCS). Bacteria tend to change its environment according to different levels of regulation and expression of genes, expression of multiple operons and stress response and sporulation and cellular motility, cell aggregation, and biofilm formation. All these levels are controlled by TCS from primarily through transcription, translations, and posttranslation of regulation of genes and also through different types of protein–protein interaction and also its virulence. TCS consists of histidine kinases, which sense the environmental signal and generate the response regulator. This process is phosphorylated by the cognate histidine kinase, and it also sometimes function as transcription regulator to regulate the expression of genes (Wang et al., 2007; Galperin, 2010). As this protein is non-homolog to human and also found to be essential and virulent, this protein could be a potential drug target against *Sg.* Table 16 presents best interacting lead molecules along with their ZINC ID, interacting residues, number of interactions, dock score, and minimized energy. Binding site residues His74, Ser114, Arg117, Lys156, and Lys153 were predicted to interact with ZINC38140720 as shown in Figure 14.

**TABLE 16:** DNA-binding response regulator and its interaction profile with docked compounds, their ZINC ID, minimized energy, number of interactions, dock score, and interactive residues.

ZINC ID	Number of interactions	Interacting residues	Minimized energy	Dock score
ZINC22108884	6	Lys 153, Lys 156, Arg 117	–27.838	–19.4464
ZINC27572262	5	Arg 117, Lys 156, Lys 153, Lys 156, Gln 140	–19.566	–20.5433
ZINC31156942	6	Gly 138, Gln 140, Arg 117, Lys 156, Lys 153	–26.579	–17.2159
ZINC17353456	7	Gln 140, Lys 156, Arg 117, Lys 153	–23.609	–17.5458
ZINC71777127	7	Lys 153, Lys 156, Arg 117, His 74, Gln 140	–30.601	–16.2028
ZINC72271115	6	Ser 114, Lys 156, Lys 153	–25.49	–20.9289
ZINC01532584	5	Lys 153, Lys 156, Arg 117, His 74	–27.704	–16.6738
ZINC01618279	5	Gly 152, Lys 153, Lys 156, Arg 117	–15.932	–14.1481
ZINC01673626	5	Lys 156, Lys 153	–29.47	–13.6307
ZINC38140720	7	His 74, Ser 114, Arg 117, Lys 156, Lys 153	–34.255	–18.0982

For each target protein, we were able to shortlist 10 lead molecules out of which 1 molecule was ranked on top. It would be appropriate to translate these *in silico* findings into *in vitro* and finally *in vivo* to channelize the computational findings toward experimental validation.

## Conclusion

In the current study, we have used an *in silico* approach in which 1,138 core proteins of 7 strains of *Sg* were determined from pan genome analysis. Subtractive genomic and identification of essential genes further reduced the number of selected targets to 18. The exploitation of 3D structural information and drug prioritization of these proteins enabled to prioritize 12 putative drug targets. All of the identified drug targets are playing an essential role in the bacterial growth, survival, and virulence, which could act as potential therapeutic targets. Furthermore, molecular docking analysis allowed us to shortlist 10 active molecules from which the best active molecule was selected on the basis of drug score, number of interactions, and binding free energy. Thus, this study provides a significant breakthrough in designing new and potent compounds against *Sg*. For the future work, the experimental validation of these targets is suggested to validate its role in survival and virulence of *Sg*.

## Data Availability Statement

The raw data supporting the conclusions of this article will be made available by the authors, without undue reservation.

## Author Contributions

NQ, RU, and SJ conceived the idea and performed the experimental work. NQ, MF, MSh, AB, HM, MSo, and SJ performed all the analysis. NQ and RM drafted the manuscript. SJ, SB, and RU critically reviewed the manuscript and provided intellectual support. All authors contributed to the article and approved the submitted version.

## Conflict of Interest

The authors declare that the research was conducted in the absence of any commercial or financial relationships that could be construed as a potential conflict of interest.

## Footnotes

1 https://www.ncbi.nlm.nih.gov/genome/

2 http://web.expasy.org/protparam/

3 https://www.uniprot.org/

4 http://cello.life.nctu.edu.tw/

5 https://www.genome.jp/kegg/

6 http://www.mgc.ac.cn/VFs/

7 https://www.ncbi.nlm.nih.gov/
